# Potential Role of Oral Microbiota in Medication-Related Osteonecrosis of the Jaw in Cancer Patients: A Narrative Review

**DOI:** 10.7759/cureus.89943

**Published:** 2025-08-12

**Authors:** Rawan J Al Harrasi, Aaisha Y Al Balushi, Fatma I Al Kindi, Nadia A Al Kindi, Amany H Kamel

**Affiliations:** 1 Dentistry, Sultan Qaboos Comprehensive Cancer Care and Research Center, University Medical City, Muscat, OMN; 2 Research Laboratories, Sultan Qaboos Comprehensive Cancer Care and Research Center, University Medical City, Muscat, OMN

**Keywords:** antiresorptive agents, cancer patients, chronic inflammation, dysbiosis, immune responses, oral health, oral microbiota, osteonecrosis

## Abstract

Medication-related osteonecrosis of the jaw (MRONJ) is a severe complication frequently observed in cancer patients undergoing antiresorptive therapies, such as bisphosphonates and denosumab. Emerging evidence suggests that dysbiosis of the oral microbiota plays a pivotal role in the pathogenesis of MRONJ. The complex interplay between microbial communities, host immune responses, and the effects of cancer treatments creates an environment conducive to pathogenic colonization, chronic inflammation, and impaired bone healing, which are the key hallmarks of MRONJ. Chemotherapy, radiotherapy, and antiresorptive agents significantly disrupt oral microbiota homeostasis, reducing microbial diversity and the overgrowth of opportunistic pathogens. These alterations exacerbate the inflammatory responses, accelerate bone resorption, and impede tissue repair. The identification of specific microbial biomarkers associated with MRONJ could facilitate early detection and targeted interventions, such as antimicrobial and probiotic therapies, to restore the microbial balance and mitigate the risk of MRONJ. Furthermore, the implementation of personalized preventive protocols, including rigorous oral hygiene and multidisciplinary collaboration among oncologists, dentists, and microbiologists, is critical for reducing the incidence and severity of MRONJ in high-risk populations. Future research should focus on elucidating the mechanisms by which microbial dysbiosis contributes to MRONJ, validating microbiome-based diagnostic tools, and optimizing therapeutic strategies to preserve oral and systemic health in patients with cancer. Integrating microbial ecology into the MRONJ management framework offers a promising avenue for addressing this challenging condition and improving the outcomes for vulnerable individuals.

## Introduction and background

Medication-related osteonecrosis of the jaw (MRONJ) is a serious complication primarily affecting cancer patients receiving antiresorptive therapies such as bisphosphonates or denosumab. Both are widely prescribed to manage bone metastases and osteoporosis, a common outcome of cancer and its treatment, contributing to improved patient outcomes [1]. However, their use is associated with a risk of MRONJ, a debilitating condition marked by persistent jawbone exposure, delayed healing, and substantial functional and aesthetic consequences [1,2]. Given its clinical burden, the prevention and management of MRONJ requires close interdisciplinary collaboration between oncologists and dental professionals [3].

Emerging evidence indicates that oral microbiota alterations are most likely to play a crucial role in MRONJ pathogenesis. The oral cavity harbors a dynamic and complex microbial community that plays a vital role in oral and systemic health. Dysbiosis refers to a significant imbalance in the oral microbiome, where beneficial bacteria are outnumbered by pathogenic microorganisms, potentially leading to adverse health conditions like MRONJ. Dysbiosis has been implicated in inflammatory conditions such as periodontitis and peri-implantitis, which share pathological features with MRONJ, including chronic inflammation and impaired tissue regeneration [4]. This overlap raises a basic question: could some alterations in the oral microbiome influence the initiation or progression of MRONJ?

Moreover, cancer treatments such as chemotherapy and radiotherapy can significantly alter the oral microbial environment, promoting opportunistic infections and pathogenic biofilm formation [5]. These disturbances may exacerbate local immune dysfunction and interact with antiresorptive agents, collectively increasing the risk of MRONJ. Identifying microbial biomarkers associated with MRONJ could therefore aid in early detection, risk stratification, and the development of microbiome-targeted interventions.

This narrative review aims to explore the role of oral microbial dysbiosis in the development and progression of MRONJ. Specifically, it examines how cancer therapies, particularly antiresorptive agents, chemotherapy, and radiotherapy, disrupt oral microbiota balance, contributing to inflammation, bone necrosis, and impaired healing. The review also discusses the potential of microbial biomarkers in guiding early diagnosis and preventive strategies to improve outcomes for at-risk patients.

Literature search strategy

This narrative review was conducted by searching major electronic databases, including PubMed, Scopus, and Google Scholar, to identify relevant studies on the role of oral microbiota in MRONJ among cancer patients. Keywords and MeSH terms used in various combinations included: "MRONJ", "oral microbiota", "microbiome", "cancer", "bisphosphonates", "denosumab", and "osteonecrosis". The inclusion period covered studies published from 2002 to 2025, with a focus on peer-reviewed original articles, reviews, and clinical or experimental studies. Reference lists of selected articles were also screened to identify additional relevant literature. Articles were selected based on their scientific relevance, methodological quality, and contribution to understanding the interaction between the oral microbiome and MRONJ pathogenesis in oncology patients.

## Review

Oral microbiota: a brief overview

The oral microbiota is a complex and dynamic ecosystem composed of bacteria, fungi, viruses, and archaea that colonize distinct niches within the oral cavity, including the teeth, tongue, gingiva, and mucosal surfaces [6]. To date, more than 700 bacterial species have been identified within the oral microbiome, with key genera including *Streptococcus*, *Actinomyces*, *Fusobacterium*, *Prevotella*, and *Porphyromonas* [7]. These microorganisms exist within a structured biofilm that interacts with the host tissues and immune responses to maintain oral homeostasis.

The composition and diversity of the oral microbiome are influenced by multiple factors such as genetics, diet, oral hygiene practices, and environmental exposure [8]. A diverse and balanced microbiome is critical for oral health, with commensal bacteria playing a protective role by producing antimicrobial peptides, competing with pathogenic species for nutrients and space, and modulating immune responses to prevent inflammation [7].

In addition to oral health, oral microbiota is intricately linked to systemic health, serving as a gateway microbiome that interfaces with the gastrointestinal, respiratory, and cardiovascular systems. Dysbiosis of the oral microbiome has been associated with systemic conditions, such as cardiovascular diseases, diabetes, and adverse pregnancy outcomes [7]. In cancer therapy, dysbiotic changes can favor the proliferation of pathogenic species, promote biofilm development, and sustain chronic inflammation, which are strongly implicated in diseases such as periodontitis, peri-implantitis, and MRONJ [9].

Given the emerging evidence linking microbial imbalances to MRONJ, understanding the role of oral microbiota in MRONJ pathogenesis is critical for developing targeted knowledge-based preventive strategies.

MRONJ pathophysiology

Despite its severity, the precise pathophysiology of MRONJ remains unclear. This condition is widely recognized as a multifactorial process influenced by pharmacological, immunological, and environmental factors [10].

Factors contributing to MRONJ

Antiresorptive Therapy

Bisphosphonates and denosumab, the two primary classes of osteoclast inhibitors, effectively reduce skeletal-related events in cancer-associated bone metastases. However, by suppressing bone turnover, these agents impair the bone’s ability to remodel and heal, particularly after dental extraction or trauma. The jawbone is especially vulnerable owing to its high turnover rate and constant exposure to microtrauma [11].

Immune Dysregulation

Both antiresorptive and cancer therapies disrupt immune homeostasis, impairing infection control and tissue repair. Immune cells, particularly macrophages and T-cells, play essential roles in bone healing and microbial defenses. Dysregulation of these responses results in prolonged inflammation, impaired angiogenesis, and tissue necrosis [12].

Oral Health Status

Poor oral hygiene, periodontal disease, and invasive dental procedures such as extractions or implant placements significantly increase the risk of MRONJ. These factors contribute to bacterial biofilm formation and microbial infiltration into the bone tissue, exacerbating inflammation and delaying healing. Dysbiosis, characterized by overgrowth of pathogenic microbes, is believed to act as a local trigger for MRONJ development [13].

Current hypotheses on MRONJ pathogenesis

Building upon our understanding of the oral microbiota's role, we now explore current hypotheses on MRONJ pathogenesis, focusing on how microbial dysbiosis may trigger or exacerbate this condition. Several interrelated mechanisms have been proposed to explain MRONJ development.

Inhibited Bone Remodeling

Antiresorptive drugs suppress osteoclast function and prevent the clearance of micro-damaged bone. This leads to impaired turnover, leaving the bone vulnerable to necrosis, particularly under mechanical stress or infection [14]. This is a widely accepted hypothesis; however, this is based on indirect evidence from animal studies and histological findings, with limited high-quality human data to confirm causation. The review supporting this claim is a narrative synthesis, not a primary study, and lacks sample-size-driven or controlled clinical evidence.

Angiogenesis Inhibition

Bisphosphonates reduce the levels of circulating vascular endothelial growth factor, a critical mediator of angiogenesis. This impairs blood vessel formation, leading to local hypoxia and nutrient deprivation, which promotes necrosis [15]. This was from an in vitro experimental study that provides strong mechanistic evidence for antiangiogenic effects of the bisphosphonate at the cellular level; the study’s limitations include its preclinical nature, lack of in vivo or human data, and focus on cartilage rather than jawbone specifically. Therefore, while the findings support the hypothesis that bisphosphonates may impair angiogenesis and contribute to necrosis, they cannot directly establish causality in clinical MRONJ without further translational or clinical evidence.

Microbial Invasion and Infection

The oral cavity harbors a diverse microbial ecosystem that serves as a reservoir for opportunistic pathogens. Once the bone is exposed, colonization of necrotic tissue by these microbes exacerbates inflammation, accelerates tissue destruction, and prevents healing [14]. This is well supported in case series, histological analyses, and animal models, with no large-scale prospective human studies demonstrating causality. As a narrative review, this remains hypothesis-generating rather than definitive, and colonization may be a consequence rather than a driver of MRONJ.

Chronic Inflammation

Persistent low-grade inflammation driven by dysregulated cytokine production plays a central role in MRONJ progression. Elevated levels of pro-inflammatory cytokines such as IL-1β, IL-6, and TNF-α have been strongly associated with impaired healing and tissue necrosis [16]. The evidence from this review study is primarily derived from animal models and in vitro studies, with no new clinical data or large human cohorts, limiting the strength of the conclusions. As a narrative review, it provides valuable insights but is hypothesis-generating rather than confirmatory, and causality remains unproven.

Oral Microbiota and MRONJ

The oral microbiota, a complex community of microorganisms, is crucial for maintaining oral health. Emerging evidence suggests that microbial dysbiosis, an imbalance in microbiota composition and function, contributes to pathogenesis [17]. Understanding the protective role of a balanced oral microbiome paves the way for examining how dysbiosis might contribute to conditions like MRONJ. Specifically, the disruption of this balance, an aftermath of cancer treatments, highlights the significance of oral microbiota in MRONJ pathogenesis.

Evidence Linking Dysbiosis to MRONJ Development

Patients with MRONJ exhibit significant alterations in their oral microbiota compared with healthy individuals, with microbial profiling revealing an increase in pathogenic species and a reduction in beneficial bacteria [18,19]. Dysbiosis is influenced by antiresorptive therapy, oral hygiene status, and necrotic bone exposure, creating an environment that favors pathogenic colonization [20].

Mechanisms by which dysbiosis impairs bone healing

Inflammation

Pathogenic bacteria within a dysbiotic environment induce excessive pro-inflammatory cytokine production (e.g., IL-1β, IL-6, and TNF-α), disrupting bone remodeling and delaying regeneration [21].

Biofilm Formation

Bacterial biofilms on exposed necrotic bones frequently characterize MRONJ lesions. The biofilm matrix protects bacteria from immune responses and antibiotics, perpetuating chronic infection and preventing epithelial and bone regeneration [14].

Direct Microbial Effects on Bone Cells

Particular bacterial species secrete virulent factors that directly affect bone metabolism. For example, lipopolysaccharides from gram-negative bacteria stimulate osteoclast activity while inhibiting osteoblast function, accelerating bone resorption, and worsening tissue damage [22].

Specific Microbial Species Implicated in MRONJ

Several bacterial species commonly associated with periodontal and endodontic infections have been linked to MRONJ. Among these, *Fusobacterium nucleatum* plays a crucial role in promoting biofilm stability and is frequently detected in MRONJ lesions. Another significant pathogen is *Porphyromonas gingivalis*, a keystone periodontal bacterium known for secreting proteolytic enzymes that disrupt extracellular matrix components. This disruption ultimately impairs both soft- and hard-tissue healing, contributing to the pathogenesis of MRONJ [4].

Additionally, *Actinomyces* species are often isolated from MRONJ lesions and are implicated in chronic infections and the formation of necrotic tissue, which can lead to non-healing wounds. Another noteworthy pathogen, *Treponema denticola*, exacerbates tissue destruction and further hinders wound healing, particularly in conjunction with other microbes. This complex interplay of bacterial infections underscores the multifactorial nature of MRONJ. It highlights the importance of understanding these microbial contributions to develop effective preventive and therapeutic strategies linked to MRONJ [4].

**Figure 1:**
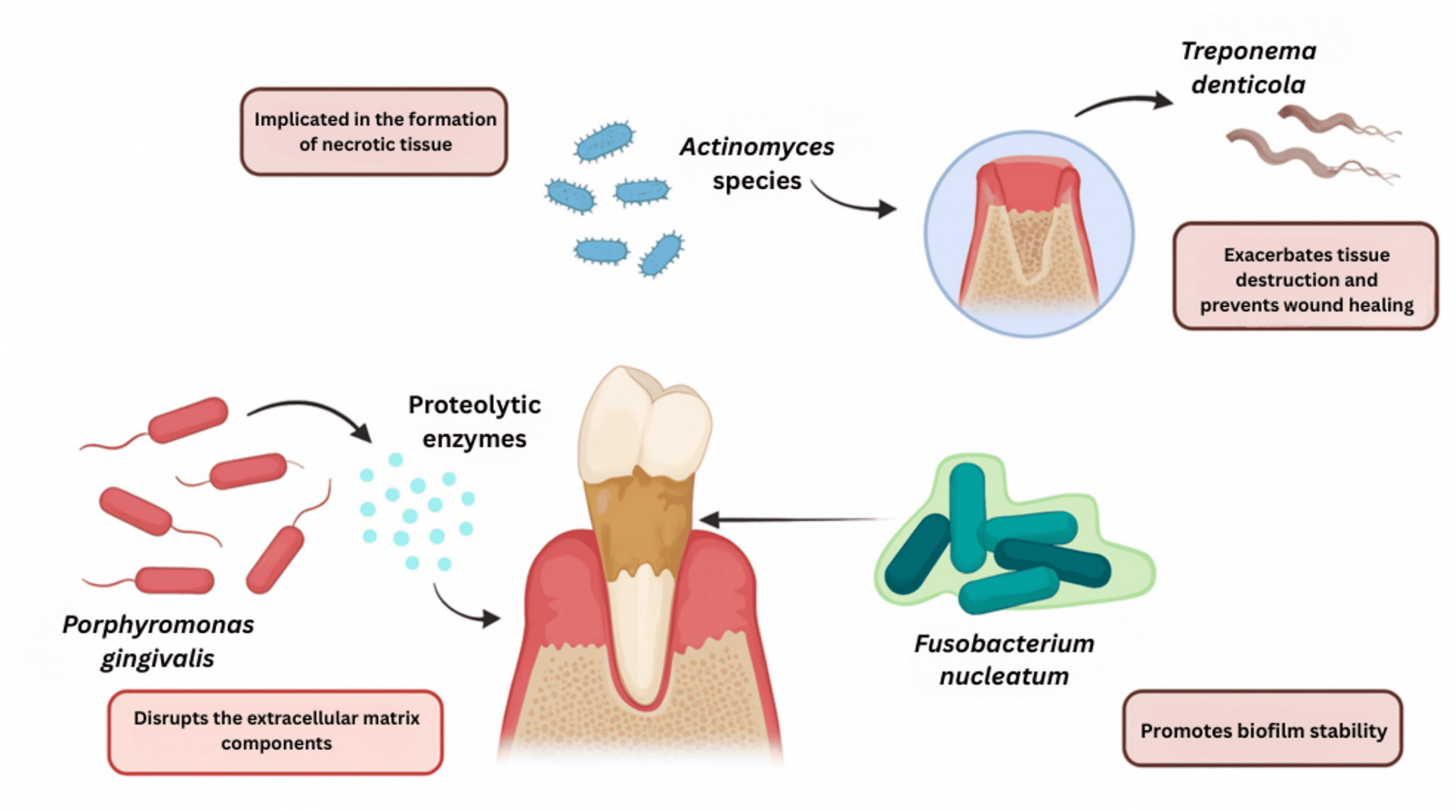
Microbial species implicated in MRONJ MRONJ: medication-related osteonecrosis of the jaw Image Credit: Authors

Interaction between cancer therapies and oral microbiota

Cancer treatment, including chemotherapy, radiotherapy, and antiresorptive agents, significantly disrupts oral microbiota homeostasis and increases the risk of MRONJ and other oral complications.

Chemotherapy

Systemic immunosuppression resulting from chemotherapy significantly impacts microbial regulation, leading to a reduction in microbial diversity and allowing opportunistic pathogens to proliferate. This alteration in the microbiome has been observed across different patient demographics, revealing distinct shifts in microbial composition [23].

In pediatric patients undergoing chemotherapy, there is a higher prevalence of specific bacterial species, particularly *Streptococcus viridans*, *Streptococcus mutans*, *Lactobacillus*, and *Capnocytophaga*. These microbial changes can have various implications for the oral health of young patients. Additionally, adult patients exhibit an increase in both gram-negative and gram-positive bacteria, with notable species including *Klebsiella* spp., *Escherichia coli*, *Enterobacter*, and *Pseudomonas* spp., as well as *Streptococcus* spp. and *Staphylococcus* spp., which contribute to serious complications such as bacteremia and antimicrobial resistance, thereby heightening the risks associated with infections. Furthermore, chemotherapy-induced conditions, including mucositis, *Candida* infection, and other secondary infections, exacerbate the microbial imbalance in the oral cavity [23].

Radiotherapy

Head and neck radiotherapy impairs salivary gland function, leading to xerostomia and reduced microbial clearance. This promotes the overgrowth of yeast and acidogenic bacteria, notably *Candida albicans*, which is detected in 54% of radiotherapy patients compared to 15% in healthy individuals [24]. Additionally, radiation-induced tissue damage facilitates biofilm formation, increasing the risk of MRONJ.

Antiresorptive Drugs

Bisphosphonates and denosumab, commonly used to manage bone metastases, alter bone turnover and create necrotic areas susceptible to microbial colonization. MRONJ-associated dysbiosis is characterized by an overrepresentation of *Fusobacterium nucleatum*, *Porphyromonas gingivalis*, and other opportunistic pathogens that impair wound healing. Furthermore, the increased prevalence of chemotherapy-resistant bacteria (*Klebsiella* spp., *Pseudomonas* spp., and *Enterobacter* spp.) exacerbates oral health deterioration during antiresorptive therapy [23].

Relationship between oral microbiota and MRONJ in patients with cancer

Microbial Colonization and Biofilm Formation in Immunocompromised States

Patients with cancer undergoing chemotherapy and radiotherapy often experience significant alterations in their oral microbiota due to immunosuppression. OPC is a common fungal infection in immunocompromised individuals, including patients with cancer, neonates, and HIV-positive individuals. In severe cases, *Candida* bloodstream infections (candidemia) result in high mortality, particularly in intensive care settings [25]. Chemotherapy-induced mucosal damage further exacerbates these infections, enabling dysbiotic microbial communities to become more virulent and invasive [25].

Cancer Treatment Regimens and MRONJ Susceptibility

MRONJ is strongly associated with chemotherapy because its immunosuppressive effects increase the susceptibility to infections. Several studies have documented the role of chemotherapy in MRONJ development, primarily through its cytotoxic effects on bone metabolism and vascularization. Certain drugs, such as thalidomide, commonly used for multiple myeloma, increase the risk of MRONJ by 2.4-fold [26]. Additionally, newer anticancer agents, particularly when combined with bisphosphonates, further elevate MRONJ risk by inhibiting tissue healing, promoting epithelial destruction, suppressing immune responses, and inducing osteoclast differentiation [26].

Pathogen Prevalence in Cancer Patients Undergoing Therapy

Cancer therapies, particularly chemotherapy and radiotherapy, drastically disrupt oral microbiota, allowing opportunistic pathogens to thrive. Patients undergoing these treatments are highly susceptible to oral infections such as oral candidiasis. Neutropenia, a common consequence of chemotherapy, facilitates the transition of *Candida* species from commensals to pathogens [27]. Moreover, both chemotherapy and radiotherapy significantly depleted nitrate-reducing bacteria, a process observed in multiple studies (34 studies), contributing to post-treatment oral complications [27].

Mechanisms linking oral microbiota to MRONJ in cancer patients

Enhanced Inflammatory Response and Delayed Healing in Cancer Patients

Cancer treatments, such as chemotherapy and radiation therapy, significantly impact inflammatory responses and wound healing, predisposing patients to MRONJ. These therapies disrupt normal tissue repair by prolonging inflammation, impairing healing, and inducing oral microbiota dysbiosis, which may act as a pro-osteonecrotic factor [13,28]. Reduced microbial diversity and overgrowth of pathogens exacerbate inflammation and bone resorption, increasing MRONJ risk, while a balanced microbiome appears to exert protective effects.

In patients with cancer, chronic inflammation leads to persistent tissue damage and impaired repair mechanisms, which may further contribute to tumor progression [29]. This prolonged inflammatory state maintains immune cell activation and inflammatory mediators at injury sites or within the tumor microenvironment, impeding healing and potentially facilitating MRONJ development.

Both local and systemic infections, along with oral microbiome alterations, influence the inflammatory status of cancer patients. The microbiome affects bone metabolism and inflammation, potentially playing a role in the pathophysiology of MRONJ. Studies have suggested that commensal bacteria can protect against inflammation-induced osteonecrosis in mice, highlighting the importance of microbiome stability in bone health [28,30]. Understanding these interactions is critical for managing delayed healing and inflammation-associated conditions in cancer patients.

Microbial-Induced Bone Metabolism Disruption

MRONJ is a severe condition frequently observed in patients with cancer receiving bisphosphonates, antiangiogenic agents, or mTOR inhibitors. Oral microbiota interacts with MRONJ pathogenesis, particularly through its effects on the ratio of receptor activator of nuclear factor kappa-B ligand to osteoprotegerin (RANKL/OPG), a key regulator of bone resorption [31]. Microbiota disturbances can influence bone metabolism, exacerbate RANKL/OPG imbalance, and contribute to MRONJ progression.

Patients with MRONJ often exhibit increased interleukin-6 (IL-6) levels and altered RANKL/OPG homeostasis, which promotes excessive bone resorption and impaired healing. Infections and microbiome alterations can further modify inflammatory responses, affect bone metabolism, and potentially contribute to MRONJ etiology. Animal studies suggest that commensal bacteria may help regulate inflammation-associated osteonecrosis, reinforcing the role of the microbiome in bone health [29]. Investigating these mechanisms is crucial for developing targeted interventions to enhance healing and mitigate the risks of MRONJ.

Impact of Chemotherapy and Radiation on Oral Microbial Ecology

Chemotherapy and radiation therapy profoundly alter the oral microbiome, leading to dysbiosis and an increased susceptibility to MRONJ. These treatments reduce microbial diversity and promote the expansion of pathogenic bacteria, triggering inflammatory responses and accelerating bone resorption [32].

One significant consequence of cancer therapy is oral mucositis, painful inflammation, and ulceration of the oral mucosa, which are all closely linked to microbiome dysregulation. The shift in microbial composition involves a decrease in beneficial bacteria and overgrowth of pathogenic species, exacerbating mucosal injury and delaying recovery. Given these effects, maintaining oral health during cancer treatment is essential for mitigating microbiome-related complications and reducing the risk of MRONJ [33].

Diagnostic and therapeutic implications for cancer patients

Microbiome-Based Biomarkers for Early Mronj Detection in Oncology

Microbiome-based biomarkers may be promising tools for early detection of MRONJ, which is critical for the successful management of affected patients, as demonstrated in several recent studies.

Kim et al. (2016) reported the oral microbiome of MRONJ patients using next-generation sequencing analysis. The researchers measured the genomes of bacteria in individuals with MRONJ and found that the bacterial communities were significantly different in patients with MRONJ than in healthy individuals. Importantly, patients with MRONJ had an abundance of bacteria related to bone resorption and bone infection. Thus, these results indicate that certain modifications in the oral microbiome may be early markers of MRONJ progression [34].

Exploring this phenomenon further in a separate study, Schwartzová et al. (2024) performed a proteomic study on the saliva of patients with MRONJ. They identified differentially expressed proteins associated with immune responses and bone metabolism [35]. The differential expression of these proteins could present a signal of changes in the oral environment occurring before MRONJ clinical symptoms, which could mark their potential as early biomarkers.

Additionally, Yatsuoka et al. (2019) conducted metabolomic profiling of saliva to identify potential early detection markers of MRONJ. They found that the level of hypotaurine in the saliva of patients with MRONJ was significantly elevated when compared to controls [36]. This metabolic product may be helpful as a biomarker for the early detection of MRONJ, which requires no invasive approach.

These studies suggest that microbiome-based biomarkers are promising for the early diagnosis of MRONJ. An approach based on changes in the oral microbiome and related biological molecules may allow healthcare providers to detect MRONJ during the stage when intervention is more effective. Further studies are needed to confirm these results and produce standardized diagnostic tools for use in clinical practice.

Adjusting Antimicrobial and Probiotic Therapies for Cancer Patients

Due to treatments such as chemotherapy and radiation, cancer patients also tend to have a weakened immune system, making them vulnerable to infections. It is fundamental to adapt antimicrobial treatments to treat these infections effectively and to limit their side effects [37]. However, overuse of broad-spectrum antibiotics can disrupt the gut microbiome, causing dysbiosis that could have negative consequences for immune responses and the efficacy of cancer treatments. Hence, judicious use of antimicrobials is necessary [38].

The adjunct use of probiotic agents has emerged as a potential strategy to ameliorate some of the adverse effects of antibiotics. Specific strains of probiotic species, such as *Lactobacillus* and *Bifidobacterium*, have been shown to restore gut microbiota balance, improve immune function, and attenuate gastrointestinal side effects of cancer treatments [39]. For example, a systematic review showed that probiotics, which could improve treatment-related gastrointestinal adverse reactions, including diarrhea and mucositis, also improve patients’ quality of life [40].

However, whether probiotics are accessible to cancer patients is not risk-free. Immunocompromised individuals may be prone to infection by probiotic organisms. Hence, the selection of appropriate probiotic strains and close monitoring of patients during therapy are essential. To maximize outcomes, tailored treatment regimens based on an individual's health status and microbiome composition are recommended [41].

Tailoring antimicrobial strategies in patients with cancer requires a balance between the effective treatment of infections and the preservation of beneficial microbiota. Probiotic supplementation appears to be a potential method to maintain this balance, but it needs to be performed with specific strains and doses to ensure safe and effective dosages. While research suggests a potential link between probiotics and bone health and oral microbiota modulation, there's currently insufficient evidence to definitively state that probiotics can prevent or treat MRONJ [42].

Personalized Oral Preventive Protocols in Cancer Care

Data up to 2023 on personalized preventive protocols in cancer care are critical for the quantitative analysis of oral hygiene practices and dental evaluations to reduce the risk of complications associated with cancer treatments [43]. Adapting these protocols to suit the particular needs of patients helps improve treatment and enhance the quality of life.

An extensive oral examination should be performed before initiating cancer therapy. Yong et al. (2022) highlighted that these assessments allow the identification and control of potential sources of infection or irritation, thereby mitigating the risk of treatment complications [44]. This preemptive step enables the development of a tailored dental treatment plan that covers individual oral health issues ahead of treatment.

Individual oral hygiene protocols tailored to cancer treatment are essential. Filippi et al. (2023) point out that proper oral hygiene is essential to prevent mucositis, xerostomia, and radiation-induced caries [43]. Specifically, the study found that the implementation of individualized care plans encompassing appropriate oral care products and methods can significantly mitigate the severity of these adverse consequences.

This allows for immediate modifications to the chosen oral care protocols if necessary. Continuous assessments allow health care providers to identify new and emerging problems promptly and ensure that preventive interventions continue to work. This adaptive strategy contributes to the maintenance of oral health and, consequently, the overall success of cancer treatment [3].

Prevention through individualized measures, including a comprehensive assessment prior to treatment, a tailored oral hygiene regimen, and systematic monitoring, is an essential component of effective cancer management. These strategies, when individualized and adjusted according to patient needs, can minimize oral sequelae, leading to better comfort and treatment completion [3].

Cancer-specific considerations and case studies

Clinical Case Reports of MRONJ in Cancer Patients With Microbial Analysis

Several case reports have implicated various microbial factors, particularly *Actinomyces* species, in the pathogenesis of MRONJ.

MRONJ developed in a patient with prostate cancer in a case report by Russmueller et al. (2016) after treatment with denosumab. Histological findings revealed *Actinomyces* colonies in necrotic bone tissue. It has been proposed that these bacteria may be responsible for MRONJ-related infections, but it is still not clear whether *Actinomyces* contributes to the pathogenesis of MRONJ. They urged additional studies to establish whether *Actinomyces* organisms provoke MRONJ or are opportunistic invaders of devitalized bone tissue [45].

Köver et al. (2023) reported on a series of prostate cancer patients who developed MRONJ after antiresorptive therapy. Necrotic bone samples or cultures from these were often culture-positive, with high counts of *Actinomyces* and *Schaalia* species. There is supportive microbiological evidence of the rescue role of these bacteria in the pathology of MRONJ. The authors recommended surgical intervention and long-term errant antibiotic therapy for these organisms and reported good clinical outcomes in all patients [46].

Zirk et al. (2019) analyzed the microbial diversity of MRONJ lesions. The most frequently identified bacteria were *Streptococcus*, *Prevotella*, *Actinomyces*, *Veillonella*, and *Parvimonas micra*. They noted that, although *Actinomyces* species were the predominant group detected, other bacteria were also assumed to play essential roles in the microbial ecology of MRONJ. The authors concluded that excision of necrotic bone diminished the diversity of bacteria present in MRONJ lesions, specifically at the base of the lesion [47].

These case reports highlight the role of microbiome analysis in the pathogenicity of MRONJ. The association between *Actinomyces* species and MRONJ lesions is quite consistent and suggests a possible pathogenic role for such species, either as primary initiators or secondary colonizers. This knowledge will help guide focused therapies, including the use of selective antibiotics, as an additive to surgical management that can improve patient outcomes.

Impact of Oral Hygiene Protocols on MRONJ Prevention in Oncology Units

A significant concern for cancer patients prescribed antiresorptive treatment is MRONJ. Clinical case studies have investigated the impact of oral hygiene protocols within oncology on the prevention of MRONJ. Ohnishi et al. (2017) conducted a retrospective study that demonstrated professional oral hygiene management as an effective preventive measure against MRONJ in patients receiving zoledronate treatment. The study delineates how participants were divided into an intervention group and a non-intervention group, the former receiving professional oral hygiene every three to four weeks, while the latter did not receive such management. The incidence of MRONJ was significantly lower in the intervention group (5%) compared to the non-intervention group (36.4%), suggesting that routine professional oral care can mitigate the risk of MRONJ [48].

Karna et al. (2018) performed a systematic review and meta-analysis that evaluated various preventive dental approaches to reduce the risk of MRONJ in cancer patients undergoing antiresorptive therapy. The analysis included six studies with a total of 2,332 patients, revealing a reduction of approximately 77.3% in MRONJ incidence following the implementation of preventive dental strategies, which encompassed detailed oral examination and appropriate dental treatment prior to the initiation of antiresorptive therapy. These authors identified limited evidence supporting certain benefits associated with preventive dental care within this high-risk cohort. Still, due to the limitations of the included studies, the evidence for such care was of low quality [49].

Shafique et al. (2024) conducted a prospective observational study examining the relationship between oral hygiene and the severity of oral mucositis in patients receiving concurrent chemoradiotherapy for head and neck cancers. The study found that patients with poor oral hygiene exhibited higher rates of moderate to severe mucositis compared to those with good oral hygiene. These findings underscore the importance of maintaining good oral hygiene and preventing complications, including mucositis, which can indirectly influence MRONJ risk [50]. Collectively, these studies highlight an essential yet under-recognized measure, both professional and personal oral hygiene protocols, in the prevention of MRONJ among oncology patients. Dental professionals must incorporate routine evaluation, prophylaxis, and patient education on maintaining oral health as part of a multimodal strategy to reduce the risk of developing MRONJ in patients undergoing cancer therapy.

Multidisciplinary Approaches in MRONJ Management (Oncologists, Dentists, and Microbiologists)

MRONJ is a multifaceted condition necessitating a comprehensive multidisciplinary approach for effective management. This approach should involve collaboration among oncologists, dentists, and microbiologists to address all aspects of MRONJ, from prevention to intervention. He et al. (2023) conducted a systematic review of nine studies, underscoring the importance of a multidisciplinary team in managing MRONJ. The study emphasizes the necessity of coordinated efforts among oncologists, dentists, and oral and maxillofacial surgeons for the thorough assessment and treatment of patients. The authors advocate for the integration of dental examinations into oncology care coordination to identify and mitigate risk factors for MRONJ [51].

In another recent study, Čebatariūnienė et al. (2024) highlight the critical role of dentists in the multidisciplinary management of patients at risk of MRONJ. This study underscores the dentist's responsibilities in assessing modifiable risk factors, developing follow-up protocols, and facilitating communication with oncologists. The treatment of MRONJ is primarily focused on prevention, with comprehensive oral examinations and patient education being essential components in managing this condition [52].

The American Association of Oral and Maxillofacial Surgeons has issued a position paper detailing a strategy for managing patients who have, or are at risk for, MRONJ. The document emphasizes the importance of a multidisciplinary oral care management approach, which necessitates collaboration among oncologists, dentists, and oral and maxillofacial surgeons to optimize care for patients with head and neck cancer. Furthermore, it highlights the necessity of conducting preventive dental evaluations and developing individualized treatment plans to mitigate the risk of MRONJ [53].

Overall, these studies highlight the importance of a multidisciplinary approach in the management of MRONJ to ensure comprehensive care for patients, addressing prevention, initial detection, and treatment strategy. An integrated approach such as this is of vital importance to patient outcomes and the reduction in incidence and severity of MRONJ in the oncology population.

## Conclusions

The manuscript addresses some of the critical gaps that highlight the importance of oral microbiota dysbiosis in MRONJ. It illustrates the interplay between the microbiota, the host's immune and inflammatory responses, and the effects of cancer therapeutics. Oral microbiota dysbiosis, in the context of chemotherapy, radiotherapy, and antiresorptive therapy, is conducive to the domination of pathogenic microbes, persistent inflammation, and inadequate healing of bone, which are typical features of MRONJ. Further research should focus on defining the role of dysbiosis in MRONJ, developing microbiome-based diagnostic tools, and designing interventional frameworks to promote oral and overall health in patients with cancer. This research contributes to the development of innovative MRONJ preventive strategies, early intervention, comprehensive patient management, and integrated care, aiming to reduce the burden of MRONJ in vulnerable populations.

Integrating microbial ecology into the framework of MRONJ management offers a promising avenue for addressing this challenging condition, emphasizing the need for continued interdisciplinary efforts to translate scientific insights into clinical practice.
